# Enabling Multicentric Participatory Disease Surveillance for Global Health Enhancement: Viewpoint on Global Flu View

**DOI:** 10.2196/46644

**Published:** 2023-09-01

**Authors:** Onicio Leal Neto, Daniela Paolotti, Craig Dalton, Sandra Carlson, Patipat Susumpow, Matt Parker, Polowat Phetra, Eric H Y Lau, Vittoria Colizza, Albert Jan van Hoek, Charlotte Kjelsø, John S Brownstein, Mark S Smolinski

**Affiliations:** 1 Ending Pandemics San Francisco, CA United States; 2 Department of Computer Science ETH Zurich Zurich Switzerland; 3 Institute for Scientific Interchange Foundation Turin Italy; 4 FluTracking Newcastle Australia; 5 Opendream Bangkok Thailand; 6 School of Public Health University of Hong Kong Hong Kong China; 7 Pierre Louis Institute of Epidemiology and Public Health INSERM Sorbonne Université Paris France; 8 National Institute for Public Health and the Environment Bilthoven Netherlands; 9 Statens Serum Institute Copenhagen Denmark; 10 Boston Children Hospital Harvard University Boston, MA United States

**Keywords:** participatory surveillance, digital epidemiology, influenza-like illness, data transfer, surveillance, digital platform, Global Flu View, program, data sharing, public health, innovative, flu

## Abstract

Participatory surveillance (PS) has been defined as the bidirectional process of transmitting and receiving data for action by directly engaging the target population. Often represented as self-reported symptoms directly from the public, PS can provide evidence of an emerging disease or concentration of symptoms in certain areas, potentially identifying signs of an early outbreak. The construction of sets of symptoms to represent various disease syndromes provides a mechanism for the early detection of multiple health threats. Global Flu View (GFV) is the first-ever system that merges influenza-like illness (ILI) data from more than 8 countries plus 1 region (Hong Kong) on 4 continents for global monitoring of this annual health threat. GFV provides a digital ecosystem for spatial and temporal visualization of syndromic aggregates compatible with ILI from the various systems currently participating in GFV in near real time, updated weekly. In 2018, the first prototype of a digital platform to combine data from several ILI PS programs was created. At that time, the priority was to have a digital environment that brought together different programs through an application program interface, providing a real time map of syndromic trends that could demonstrate where and when ILI was spreading in various regions of the globe. After 2 years running as an experimental model and incorporating feedback from partner programs, GFV was restructured to empower the community of public health practitioners, data scientists, and researchers by providing an open data channel among these contributors for sharing experiences across the network. GFV was redesigned to serve not only as a data hub but also as a dynamic knowledge network around participatory ILI surveillance by providing knowledge exchange among programs. Connectivity between existing PS systems enables a network of cooperation and collaboration with great potential for continuous public health impact. The exchange of knowledge within this network is not limited only to health professionals and researchers but also provides an opportunity for the general public to have an active voice in the collective construction of health settings. The focus on preparing the next generation of epidemiologists will be of great importance to scale innovative approaches like PS. GFV provides a useful example of the value of globally integrated PS data to help reduce the risks and damages of the next pandemic.

## Overview

Digital epidemiology has expanded significantly in recent years [1,2], encompassing not only passive digital disease detection [3], but also active crowdsourcing programs known as participatory surveillance (PS) [4-7]. PS has been defined as the bidirectional process of transmitting and receiving data for action by directly engaging the target population [8]. Often represented as self-reported symptoms directly from the public, PS can provide evidence of an emerging disease or concentration of symptoms in certain areas, potentially identifying signs of an early outbreak. The construction of sets of symptoms to represent various disease syndromes provides a mechanism for the early detection of multiple health threats [9-12]. In addition to sets of syndromes, PS benefits from the acquisition of data on nonsymptomatic individuals, providing some measure of a denominator to make calculations on prevalence, incidence, attack rate, among other metrics [13]. It is also possible to include questions that act as proxies for the identification of risk factors that may be associated with a health threat within a community. For example, questions about seeking health care after reporting symptoms may reveal the severity of the event for those individuals [11-13]. Or asking if a participant reporting into the system has recently traveled may indicate that symptoms are from a disease acquired from another location. Inquiring whether the reporter had recent contact with someone with similar symptoms might reveal a possible contagion network [13]. Additional questions about sociodemographics (eg, age, gender, income, and occupation) or even vaccination status can complement the syndromic information provided by users who participate in such programs to help inform at-risk groups [14]. Another layer that may be emphasized around the benefits of PS is its stability when something goes epidemic. While other surveillance systems rely on the health care system and are not scalable, web-based surveillance remain standing, even when the health care system is flooded. It is very important to have reasonable stable time trends to interpret what is going on. Furthermore, not so much the exact questions of the surveillance is important, but that the system is reasonably flexible including different questions, and that you can survey whatever is necessary, whether it is the uptake of a COVID-19–tracking app or self-tests, or vaccine-sentiment.

By these evidences it seems that PS is an affordable and effective proxy for public health departments to estimate, for example, influenza-like illness (ILI) and COVID-19–like illness numbers, despite the fact that clinical surveillance efforts are receiving far less data.

After the H1N1 pandemic emerged in 2009, PS for ILI became vital for monitoring this health challenge [6,15,16]. Since then, several platforms have expanded around the world seeking to use the participation of citizens in the systematic and periodic collection of symptoms related to influenza [9,17-26]. The numerous programs monitoring ILI have provided evidence regarding the acceptability, reliability, promptness, accuracy, and usefulness of this approach [6,20,22]. On the other hand, the aspects of data integration and interoperability between these systems for the purpose of comparing ILI activity across countries and regions remain a challenge. While several programs have similar objectives, the different governance and data compliance procedures placed operational hurdles to integrate and promote interoperability between the databases. A solution to realize the value of PS in monitoring global ILI activity was desperately needed.

In 2018, the first prototype of a digital platform to combine data from several ILI PS programs was created [27]. At that time, the priority was to have a digital environment that brought together different programs through an application program interface, providing a real-time map of syndromic trends that could demonstrate where and when ILI was spreading in various regions of the globe. After 2 years running as an experimental model and incorporating feedback from partner programs, Global Flu View (GFV) was restructured to empower the community of public health practitioners, data scientists, and researchers by providing an open data channel among these contributors for sharing experiences across the network. GFV [28] is redesigned to serve not only as a data hub, but also as a dynamic knowledge network around participatory ILI surveillance by providing knowledge exchange among programs [28]. The goal of this study is to serve as a viewpoint exploration, dissecting the challenges, solutions, and potential of PS applied in digital ecosystems like GFV.

## Connecting ILI Programs Across the Globe in GFV

GFV is the first ever system that merges ILI data from more than 8 countries plus 1 region (Hong Kong) on 4 continents for global monitoring of this annual health threat. GFV provides a digital ecosystem for spatial and temporal visualization of syndromic aggregates compatible with ILI from the various systems currently participating in GFV in near real time (Table 1), updated weekly. In addition, GFV presents a list of features that support using PS as a data layer for enhanced ILI surveillance (Table 2). It is important to emphasize, however, that PS does not aim to replace traditional surveillance systems but rather to complement the information obtained from such systems and to generate actionable data.

**Table 1:** List of programs that are participating in GFV^a^ as of December 2022.

Program’s name	Territory coverage	Year of foundation	Description	Outcomes	Host institution
Outbreaks Near Me (formerly Flu Near You) [29]	United States and Canada	2011	A system that allows its users across North America to securely and anonymously self-report ILI^b^ symptoms or to report feeling healthy.	Since users generally report before visiting a health care provider, trends on the spread of ILI are often detected before local and national public health agencies do.	Boston Children’s Hospital—Harvard University
FluTracking Australia [30]	Australia	2006	A web-based community-based acute respiratory illness surveillance system that is not biased by health seeking behavior, clinician testing practices, or differences in jurisdictional surveillance method.	To contribute to community-level acute respiratory illness surveillance in Australia To provide consistent surveillance of acute respiratory illness attack rates and testing across all jurisdictions and over time. Provide year-to-year comparison of the timing, attack rates, and seriousness of acute respiratory illness in the community.	Hunter New England Local Health District, Hunter Medical Research Institute—The University of Newcastle
FluTracking Hong Kong [31]	Hong Kong	2021	A web-based health surveillance system used to detect the potential spread of influenza and COVID-19.	To compare ILI syndrome proportions according vaccination status and seasonality, providing consistent monitoring of influenza surveillance activity.	School of Public Health—the University of Hong Kong
SickSense [32]	Thailand	2020	A network of digital volunteers who participate through anonymous reporting of their own health.	This system can identify early signs of an emerging epidemic threat, be an assistant to find real time signs of illness, and engage through digital tools.	The Health Promotion Foundation and Open dream
InfluenzaNet^c^ [33]	Italy, France, the Netherlands, and Denmark	2009	A web-based survey tool to conduct syndromic surveillance through self-reported symptoms volunteered by participants residing in the InfluenzaNet countries.	The platforms collect demographic and risk-factor data from participants upon enrollment, capture weekly symptoms, and report analyzed surveillance results. This provides insights about the ILI activity.	Influweb.org, Covidnet.fr, Influmeter.dk, Infecieradar.nl

^a^GFV: Global Flu View.

^b^ILI: influenza-like illness.

^c^InfluenzaNet integration is still going on. This is a partial list of countries which enrolled in the platform.

**Table 2:** Overview of functionalities and main features of GFV.^a^

Feature	Description	Technical specification	Availability
Time-series report (Figure 1)	Temporal distribution of participant’s reports and ILI^b^ proxy defined by each program. In this feature,^c^ it is also possible to filter by specific symptoms, vaccination status, gender, and age range.	Apex charts.JS is an open-source library for AngularJS that enables a smooth user experience loading time-series charts.	Publicly available. For registered partners, download of the line listing data is also possible in .CSV format.
Map (Figure 2)	Spatial distribution of participant’s reports and ILI proxy defined by each program. In this view, it is possible to visualize in a (1) cluster view, (2) disaggregated reports by the minimum allowed level (eg, postcode centroids), or (3) heatmap with ILI cases.	For the use of clustering and heatmap view, Mapbox has been used.	Publicly available.
Resources	Library of recent peer-reviewed publications about PS.^d^	Automated scrapper that is connected with PubMed database, looking for keywords associated with PS. Before a paper is displayed on the resource page, a human input is necessary to accept or decline the suggestion. Once accepted, it populates a resource section.	Publicly available.
API^e^ documentation (Figure 3)	The documentation for API requests of postprocessed data points in the platform. The validation is based on a token generated by each program partner and the authorization is given by passing the key via header. The available requests for postprocessed data are: GET /api_map (returning data pinned to the map); GET /map_dates (returning the dates from the registered data); and GET /surveys (returning chart data from Reports section).	For the map data: Lat (number $double); Lng (number ($double)); Report_count (integer); ILI_count (integer); No_symptoms_count (integer); Some_symptoms_count; (integer); Postcode (string); Country (string); ILI_percentage (integer); No_symptoms_percentage (integer); Some_symptoms_percentage (integer); For the survey data: Match_count (integer); Percentage_count (number); Total_count (integer); Week (string)	Restricted to program partners with approved access to this feature.
Codelab (Figure 4)	Digital space for sharing codes, notes, and snippets across the community of program partners. In addition, this brings the possibility to specify the permission (whether creative commons or GPL^f^) and the language used. The codes are visible for all program partners that may use them for their projects or data analysis pipelines.	Besides the title, description, and tags, one can define the permission that the code has as well as the language the snippet was coded in.	Restricted to program partners with approved access to this feature.

^a^GFV: Global Flu View.

^b^ILI: influenza-like illness.

^c^When available by the program partner.

^d^PS: participatory surveillance.

^e^API: application program interface.

^f^GPL: General Public License.

**Figure 1:**
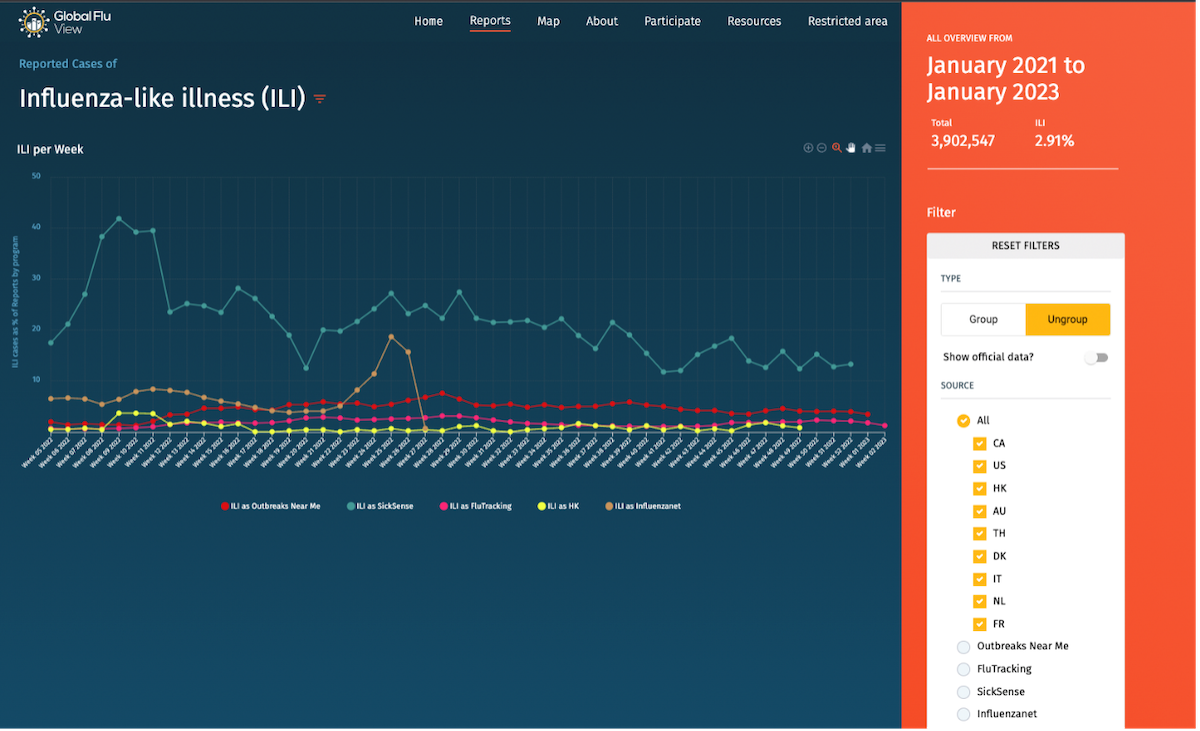
Time-series reports view, showing the aggregated number of reports and proportion related to ILI reports. The filter functionality selects specific countries or programs as well as vaccination status, age range, and gender of participants. ILI: influenza-like illness.

**Figure 2:**
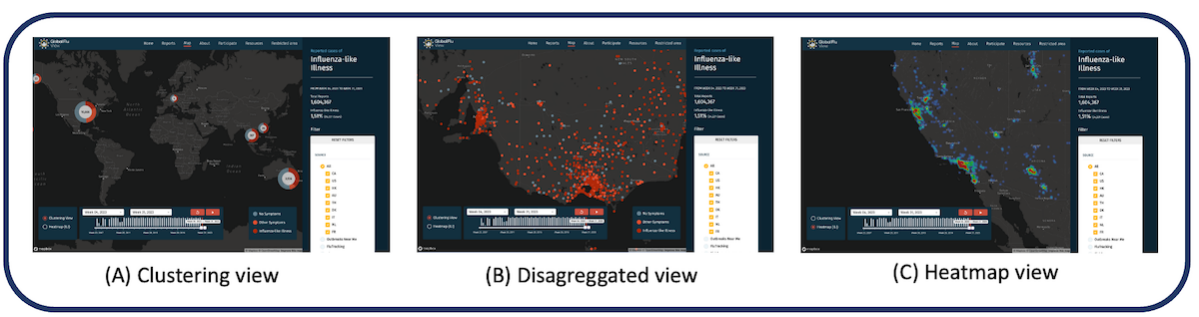
Screenshot from the map view. It shows three different views that are available for users: (A) clustering view (aggregated mode based on the zoom level); (B) disaggregated view using the centroid of a zip or postal code; and (C) heatmap view for ILI reports from the filtered period. The map view also provides visualization of clustering view, aggregating reports based on the zoom level. Disaggregated level view is supported if the partner program has given permission. ILI: influenza-like illness.

**Figure 3:**
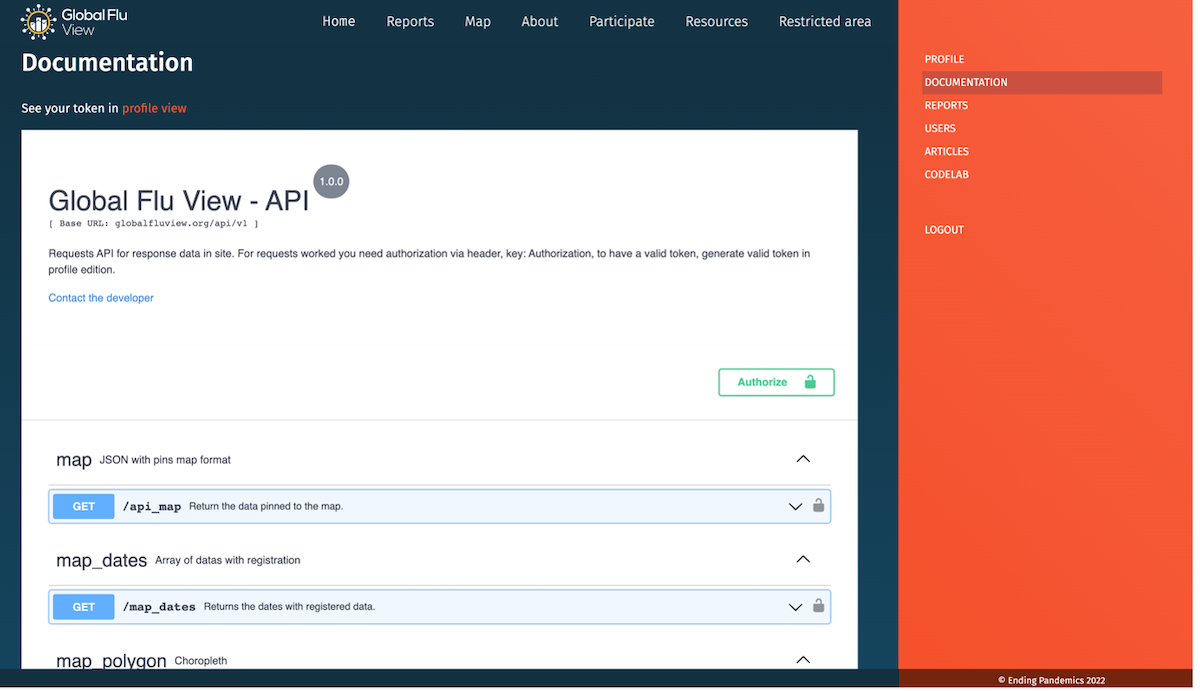
Documentation view of GFV API, providing program partners with all information needed to integrate GFV data into their systems, respecting the governance of each program and data sharing agreement. API: application program interface; GFV: Global Flu View.

**Figure 4:**
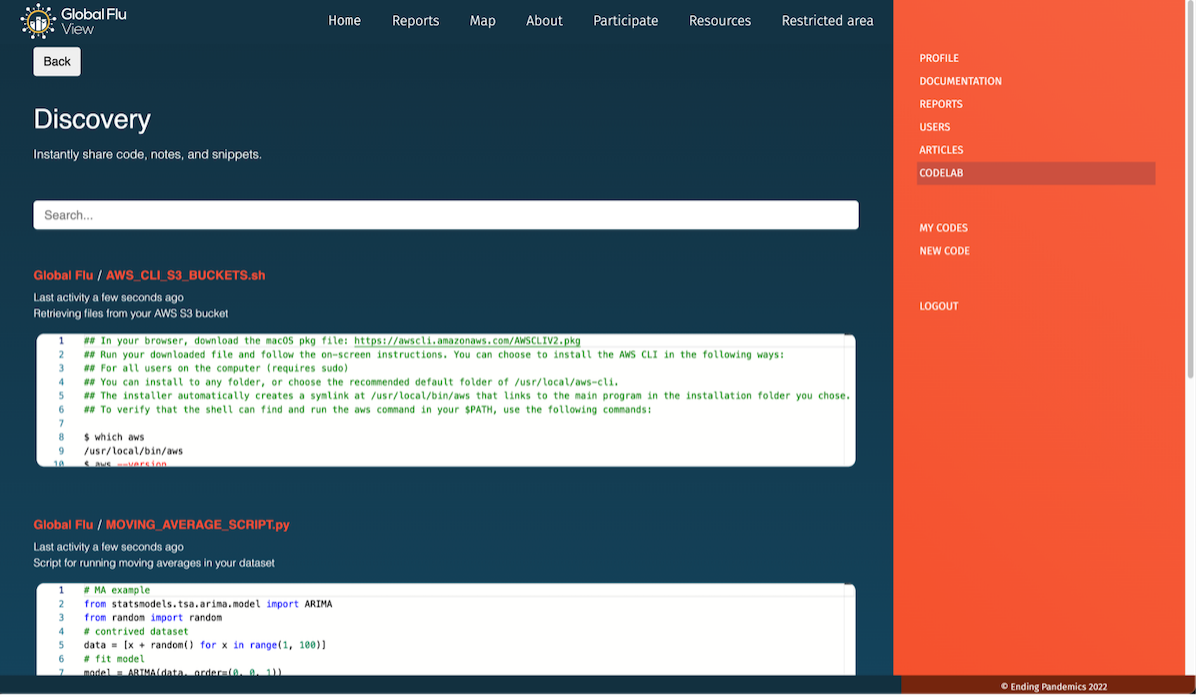
Codelab is the tool into GFV that allows program partners to share among themselves notes, scripts, and codes about techniques and methods for PS data analysis. GFV: Global Flu View; PS: participatory surveillance.

## Understanding the GFV Ecosystem

GFV may be best described as an ecosystem with three different layers: (1) program partners, (2) community, and (3) outcomes. PS programs for ILI were informed about the purpose of GFV, and after accepting the data sharing agreement, became part of the core layer by contributing to the availability of real-time data. In the second layer, the “community” of potential consumers of this data can be described in three categories: (1) public health practitioners focused on disease surveillance, (2) data consumers focused on the use and integration of the data for third-party systems and platforms, and (3) researchers, who may use the data for comparative, predictive, or characterization of global epidemiological scenarios about ILI outbreaks as well as the development of forecasting and modeling techniques. Finally, the outcomes layer demonstrates the impact that these data can generate on the knowledge of the current and future ILI landscapes; and the potential of information sharing for constructing and strengthening of the ILI PS community. Pooling data from different regions and integrating them into GFV generates a pivotal tool for participatory surveillance. The collaborative network formed from this international data integration not only fosters shared learning but also strengthens ties between researchers and public health practitioners globally. This community aspect provides a robust response to ILI on an international scale.

The global perspective offered by GFV is especially valuable considering the nature of influenza epidemics. Tracking these patterns across various geographical regions provides critical insights into disease spread dynamics, contributing significantly to our understanding of these epidemics. Further, the collective data from diverse sources amplify the statistical power of analyses conducted using this platform. With a larger, more diverse data set at their disposal, researchers can draw more robust conclusions and gain key insights into influenza trends and variations across different settings. This enriched perspective provides a significant contribution to the global understanding and management of ILI.

**Figure 5:**
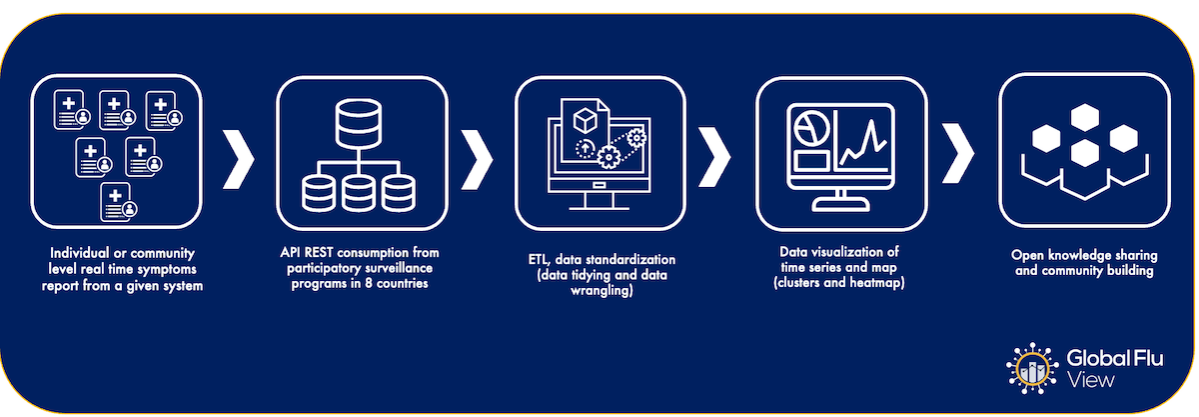
GFV’s workflow, describing the steps for data acquisition, API integration, data processing, data visualization, and knowledge sharing among program partners. API: application program interface; AWS: Amazon Web Services; ETL: Extract, Transform and Load ; GFV: Global Flu View.

The list of program partners is periodically updated, giving opportunities for ILI programs from anywhere to be part of this ecosystem. GFV is also open to the general public, who can use the potential of this tool to understand the importance of their contribution in reports to program partners in the collective construction of information on global health. Figure 5 describes the GFV’s workflow for a better understanding. We will further delve into the intricacies of community building, interoperability challenges, and data governance specific to participatory surveillance in subsequent sections. These important aspects help paint a comprehensive picture of our work and elucidate the significant contribution of GFV to the field of public health surveillance.

## Community Building and Knowledge Sharing

A community of PS enthusiasts can be connected with knowledge sharing in several ways. First, this community can provide a forum for practitioners to share their knowledge and expertise with each other. This can take the form of discussions and collaboration on specific projects or challenges, as well as the sharing of resources, such as research papers, technical manuals, and training materials. By sharing knowledge within the community, practitioners can learn from each other's experiences and can help to advance the field as a whole. Additionally, a community of health technology practitioners can also facilitate the sharing of knowledge with other stakeholders, such as policy makers, health care organizations, and the general public. For example, a community can organize conferences, workshops, and seminars where practitioners can present their research and findings to a broader audience. This can help to raise awareness of the latest developments in health technology and can encourage the dissemination of new ideas and best practices.

The GFV ecosystem aims to strengthen the use of surveillance data among professionals in various fields. Professionals such as public health practitioners, epidemiologists, data scientists, and software developers may use the GFV data in the routine of disease surveillance. The platform encourages contribution of data analysis techniques, methods, and processes to promote knowledge sharing among partners and ensures that data are used in a reproducible manner.

## Interoperability Challenges: Overcoming Hurdles Based on Data Standards

Interoperability for participatory disease surveillance data can help to ensure that data from different systems can be easily exchanged and integrated, leading to more effective and efficient processes. However, several challenges must be considered to achieve this goal, including the following: (1) lack of standardization: different PS programs may collect different types of data, at different granularities, use different data formats and structures, which can make it difficult to exchange data. Moreover, without standardized data definitions and formats, it can be difficult to ensure that data from different PS programs are comparable; (2) complexity of data integration: integrating data from diverse PS programs systems can be a complex and time-consuming process, especially if the systems are not designed to interoperate; (3) limited technical capacity: some PS systems may not have the technical capacity or expertise to integrate data from other systems; and (4) privacy and security concerns: ensuring the privacy and security of sensitive health or disease data is a key concern when integrating data from distinct surveillance systems and are subject to different national or international regulatory frameworks (eg, European Union General Data Protection Regulation).

To overcome these challenges, it is necessary to adopt data standards that provide a common framework for the exchange and integration of data from different surveillance systems. Data standards offer a set of rules and guidelines for defining data elements and formats, which can help to ensure that data from different systems are comparable and can be easily integrated.

In developing GFV, the number of global platforms that use secondary epidemiologic data was considered. Documenting the processes and end points for accessing data on the platform is fundamental to the interoperability of this data with other data streams to allow connections for integration with GFV. Using an API to connect the various systems with GFV was deemed the most efficient mechanism.

## Data Governance: Getting Legal Agreements for a Multicentric PS Ecosystem

A data sharing agreement is required for any partner that provides its data to GFV (except for those programs which make the data publicly available). This agreement covers core principles such as (1) preserving data privacy by sharing deidentified data, (2) maintaining data integrity, and (3) respecting the governance structure and compliance required by each program partner.

Data governance is an essential aspect of PS systems to safeguard the integrity, quality, and security of data collected. It includes policies, processes, and practices for data collection, management, and use, with defined roles and responsibilities for data quality assurance and security. Clear and transparent policies and practices can ensure that participants feel confident that their data will be used responsibly.

## Looking to the Future

### Enabling PS for Digital Public Health Intelligence

The popularization of social media, added to the wave of “big data,” creates the opportunity for crowdsourcing participatory disease surveillance in GFV to expand the understanding of ILI and its impact across the globe [19,20].

Several initiatives are integrating diverse data streams to increasingly provide timely information to detect public health events. Low- and middle-income countries, in particular, are greatly benefited by these approaches. Examples of these initiatives include the Integrated Disease Surveillance and Response framework in sub-Saharan Africa. For 20 years it has demonstrated the importance of improving the detection and response of public health threats by combining data from diverse systems and laboratories [34]. In China, another model framework integrates multichannel surveillance, data analysis for early warning, and a specific strategy for sustainability of the surveillance system [35]. Within the scope of PS, it is possible to see the integration of laboratory data for the monitoring of SARS-CoV-2 antibodies seroconversion [36-38]. PS has historically also used data from social media to leverage its sensitivity and detection capability [39,40]. In Netherlands, a strategy to combine PS reports plus self-tests given free of charge and centralized testing of nose or throat samples has been performed, showing another angle of empowering public health intelligence by these approaches [41]. The previous examples show how different ecosystems across the globe are ready to integrate diverse data streams, where GFV may be one of these, enabling a feasible view for PS.

During the first years of the COVID-19 pandemic, the value of other types of data, from wearables, wastewater, and mobility through cellular data for early identification of risks, became evident [42-45]. Approaches like this also show the ability of omnichannel surveillance for other infectious diseases with pandemic potential, such as a novel strain of influenza. Indeed, challenges of integration and interoperability between these data sources will exist and demand that the next generation of epidemiologists be familiar with the format and structure of each data source. The assessment for data standards for collecting comparable data will be crucial to guarantee the unity of such different systems.

### Cross-Engagement

Cross-engagement is a term inherited from the marketing industry, which describes the use of multiple marketing channels to seamlessly leverage retention of a target audience [46]. Collaborative platforms can benefit from this concept, as the retention and engagement of users in PS strategies has always been a point of friction. Maintaining engagement and participation in these programs can be challenging, as individuals and communities may have other priorities or may lose interest over time. With cross engagement, the user is motivated on multiple fronts for multiple purposes. The GFV platform provides cross-engagement as user data are seen at a local or national level by the partner platform and at an international level displaying trends for multicentric comparison between countries and continents. The empirical evidence is that people become further incentivized by the knowledge that their data matter both locally and globally.

Programs for monitoring and combating chronic diseases [47] can serve as valuable models for infectious disease PS programs, particularly for influenza, which is a key focus of the GFV platform. These programs can provide valuable insights and lessons learned that can be applied to the design and implementation of infectious disease surveillance programs, helping to ensure that these programs are effective and efficient. By leveraging the experience and expertise of chronic disease surveillance programs, it is possible to learn how to improve the engagement and retention performance of participatory infectious disease surveillance programs [48-50].

### Smart Health Communities

Smart Health Communities has been another important trend to consider in the future of disease surveillance [51]. The core features of this approach are (1) empowering individuals to proactively manage one’s own health and well-being, (2) fostering a sense of community and belonging, (3) using digital technologies and behavioral sciences, (4) using data for substantial improvements in health outcomes, and (5) creating innovative ecosystems [52].

In this scenario, studies demonstrate how these programs reach the status of social contagion to achieve a collective good [53-55]. This type of social influence can be one of the ways to increase user participation in PS platforms, considerably increasing the potential for using the data that circulates in this environment [53,54]. The parallelism between geographic smart health communities and digital smart health communities enables the possibilities of using the metaverse for PS [55]. By these examples, having a web-based hub that serve as stage for smart health communities look at the data seems realistic and needed. With the open access that GFV promotes through temporal and spatial data, this becomes a concrete example for future interactions in smart health communities.

### Opportunities for the Next Generation of Digital Epidemiologists

Given these perspectives, the importance of the next-generation of digital epidemiologists actively participating in the ongoing digital transformation of public health surveillance cannot be overstated. Their role in this rapidly evolving landscape goes beyond merely applying existing tools and systems; they must also acquire a comprehensive understanding of this emerging field. In order to cultivate a new breed of professionals capable of steering this digital revolution, the inclusion of data science, software engineering, mathematical modeling, and data design in academic and technical training programs is vital. This multidisciplinary approach equips future digital epidemiologists with the broad spectrum of skills they require to be self-reliant and innovative in their roles. While collaboration and cross-pollination of ideas are indeed essential in this multifaceted field, it is equally important for these professionals to have the capability to make independent, informed decisions. The interdependence inherent in such a diverse field should not lead to an overreliance on others. These professionals will need to be well-versed in all aspects of digital epidemiology, from disease surveillance to the intricacies of digital public health. GFV is pioneering in this educational journey by providing a platform that fosters collaboration, knowledge exchange, and the cultivation of skills. The GFV Codelab, for instance, is an exemplary feature that demonstrates how digital platforms can be harnessed as digital learning environments. As we navigate this new digital landscape, it is incumbent upon us to ensure that the next generation of professionals is well-equipped to lead the field of digital epidemiology into the future.

Further, the endorsement of participatory surveillance strategies by major health organizations, such as the World Health Organization [56], underscores the pivotal role that this approach plays in global health. It signals an expectation for the next generation of digital epidemiologists to not only understand these approaches but to implement them effectively and innovatively.

## Limitations

GFV has several limitations. First, the data in GFV are dependent on the quality of its partners’ data. Since PS programs rely on the active involvement of individuals and communities, GFV data are impacted by this reality that not all individuals and communities may be willing or able to participate in these programs. This can lead to incomplete or biased data, which can impact the accuracy and usefulness of the information collected through these programs. Second, data quality and integrity are an issue due to the characteristics of self-reported data, which can be subject to errors, biases, and inconsistencies. Third, the various programs contributing data to GFV often require significant resources, including funding, staff time, and technology. These resources may be limited, which can impact the ability of these programs to effectively collect data on an ongoing basis and share it with GFV.

## Conclusions

Connectivity between existing PS systems enables a network of cooperation and collaboration with great potential for continuous public health impact. Thus, it is crucial for professionals engaged in ILI studies, educators, students, and public health practitioners to engage with digital ecosystems. It is an opportunity to make use of these open data channels and contribute to knowledge, and help to enhance the depth and breadth of ILI surveillance. Moreover, it offers a platform for learning, interaction, and collaboration, fostering a more integrated and proactive approach toward public health challenges. The exchange of knowledge within this network is not limited only to health professionals and researchers, but also provides an opportunity for the general public to have an active voice in the collective construction of health settings. The focus on preparing the next generation of epidemiologists will be of great importance to scale innovative approaches like PS. GFV provides a useful example of the value of globally integrated PS data to help reduce the risks and damages of the next pandemic.

## Abbreviations

GFV: Global Flu View
ILI: influenza-like illness
PS: participatory surveillance
